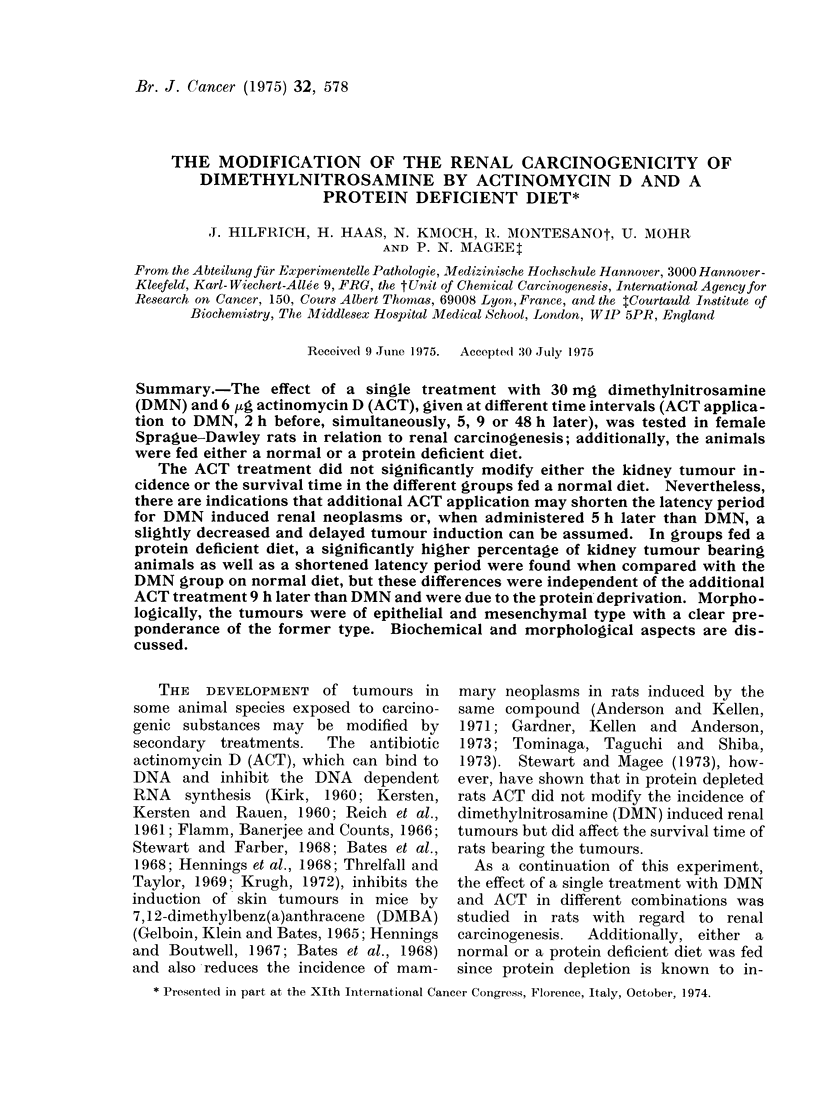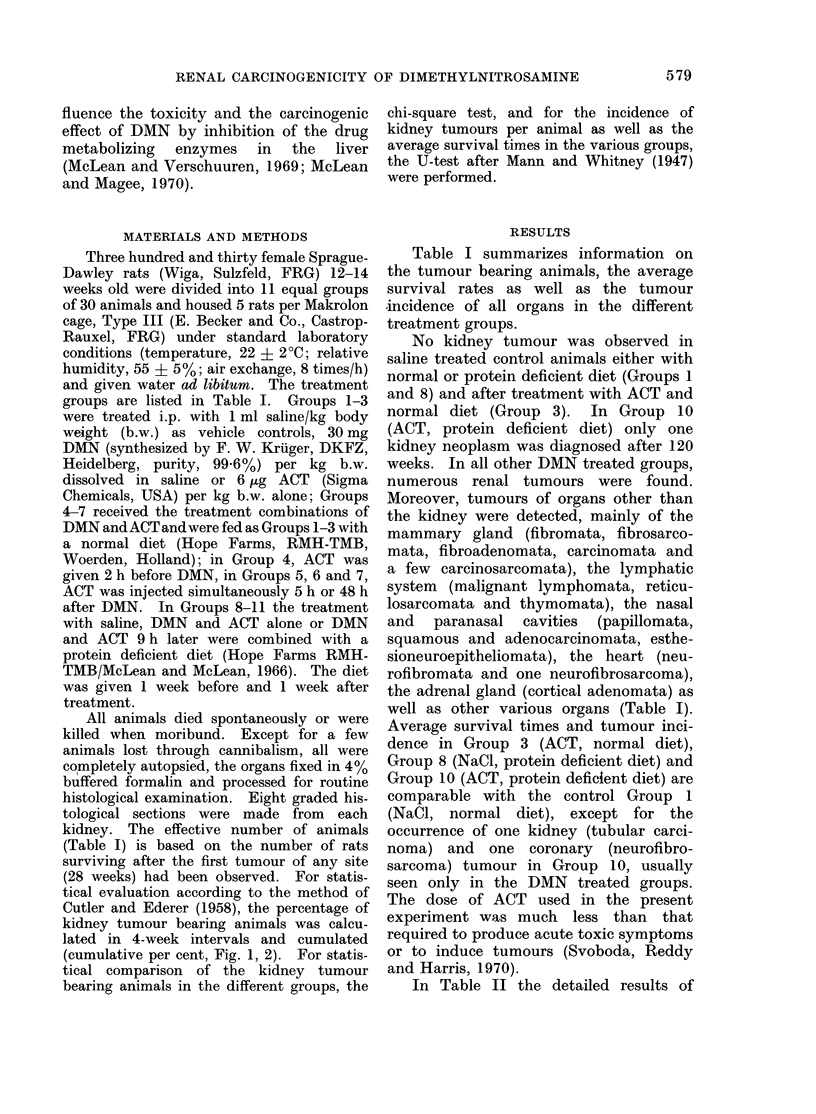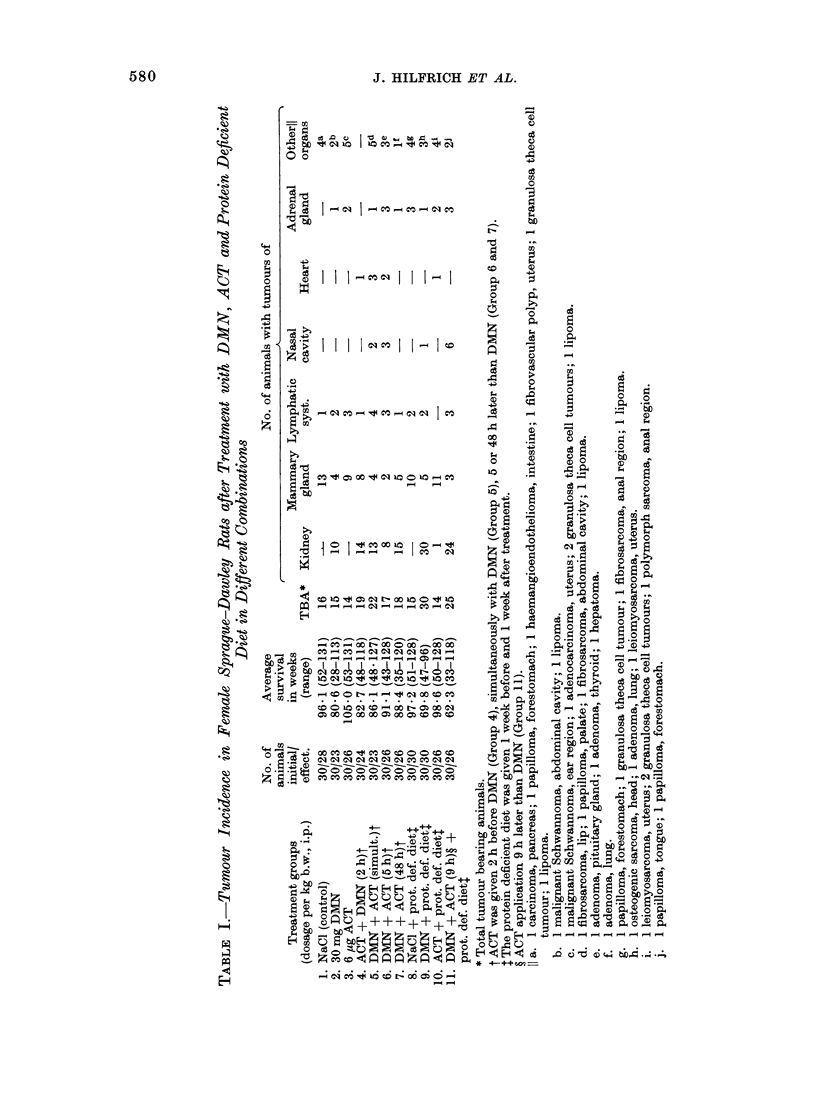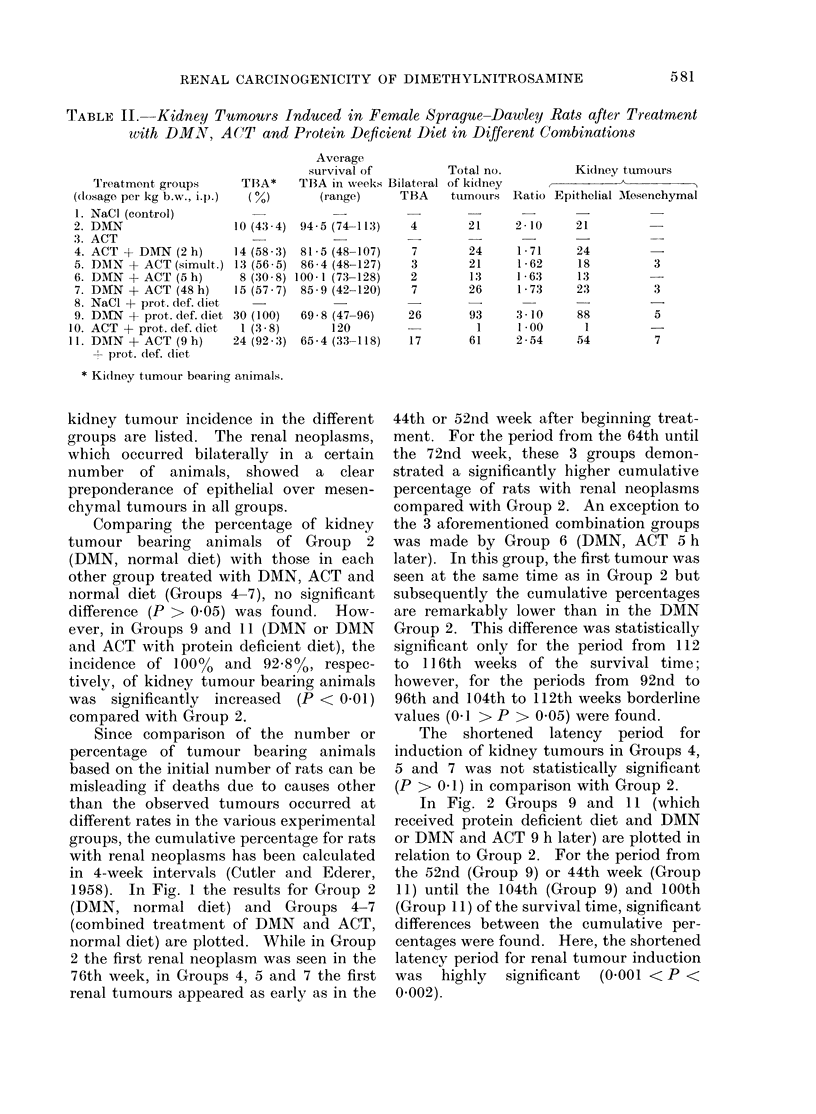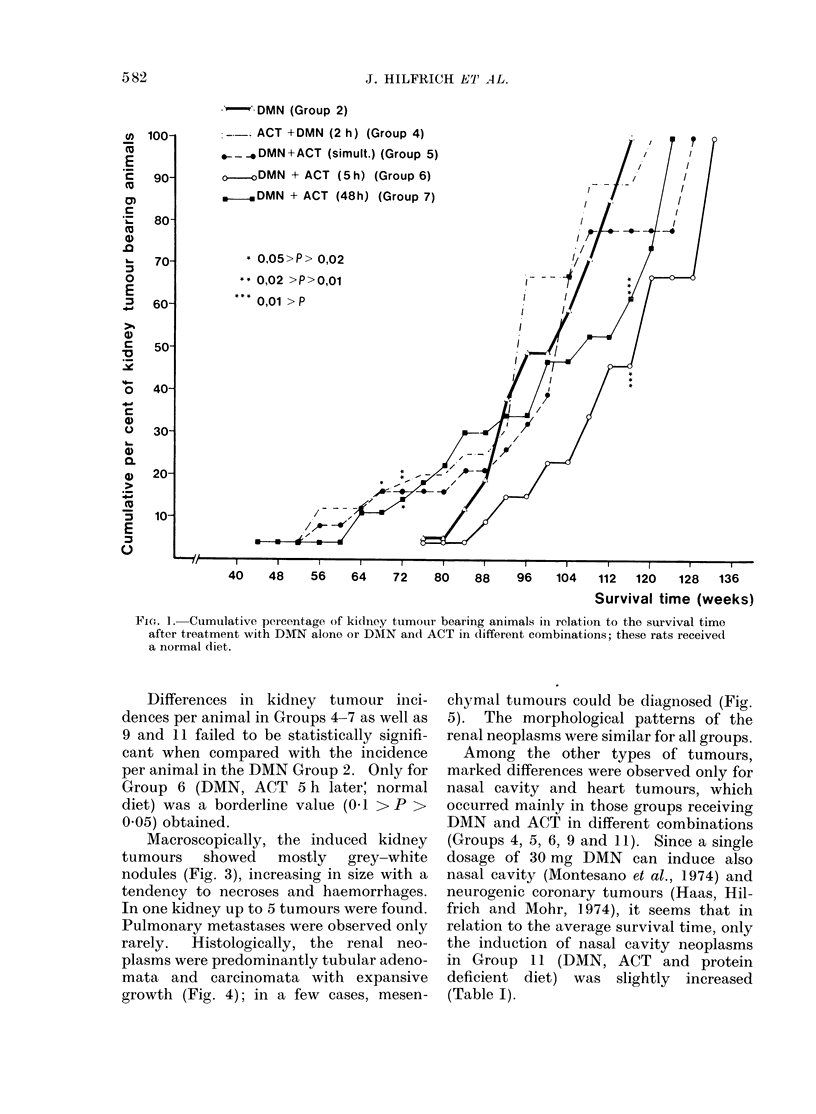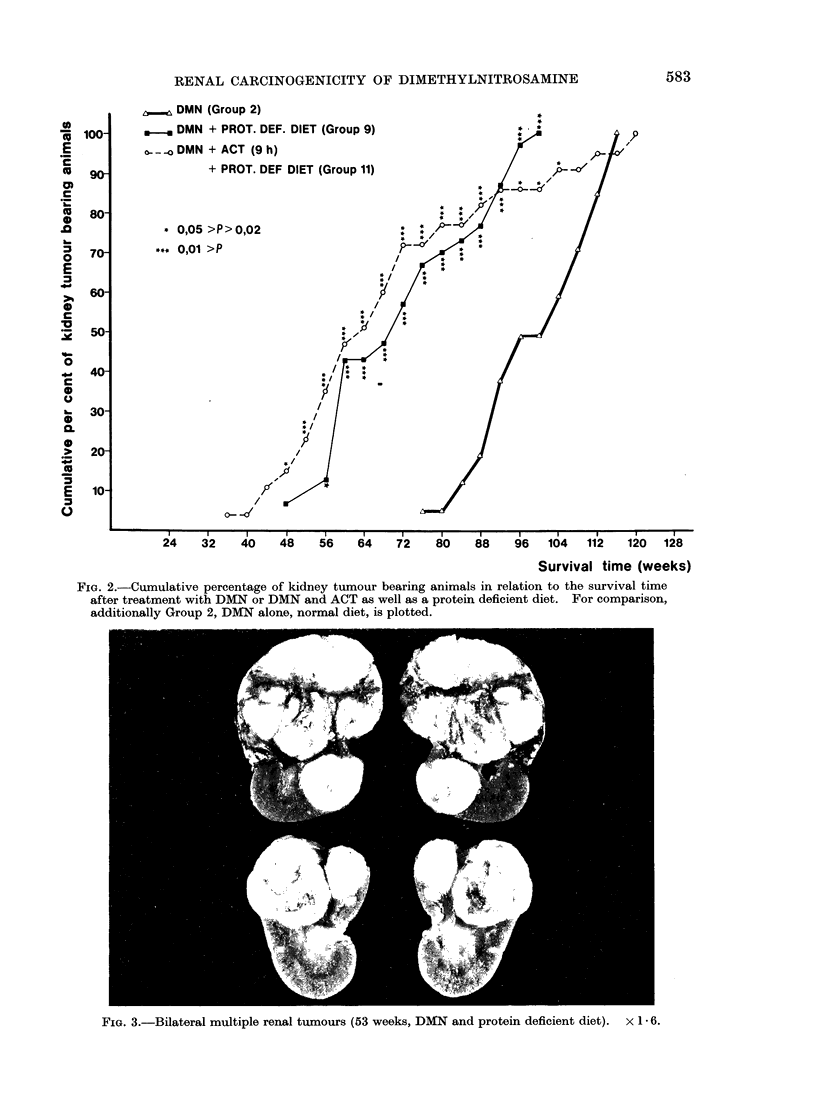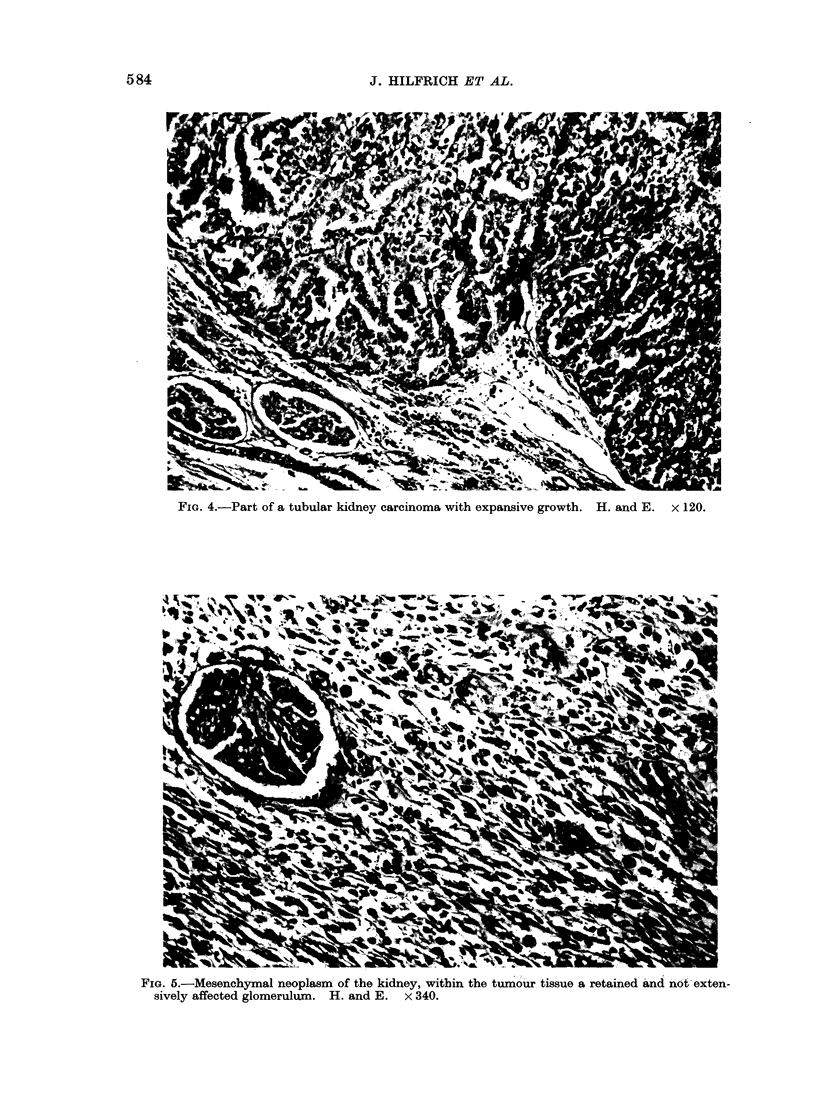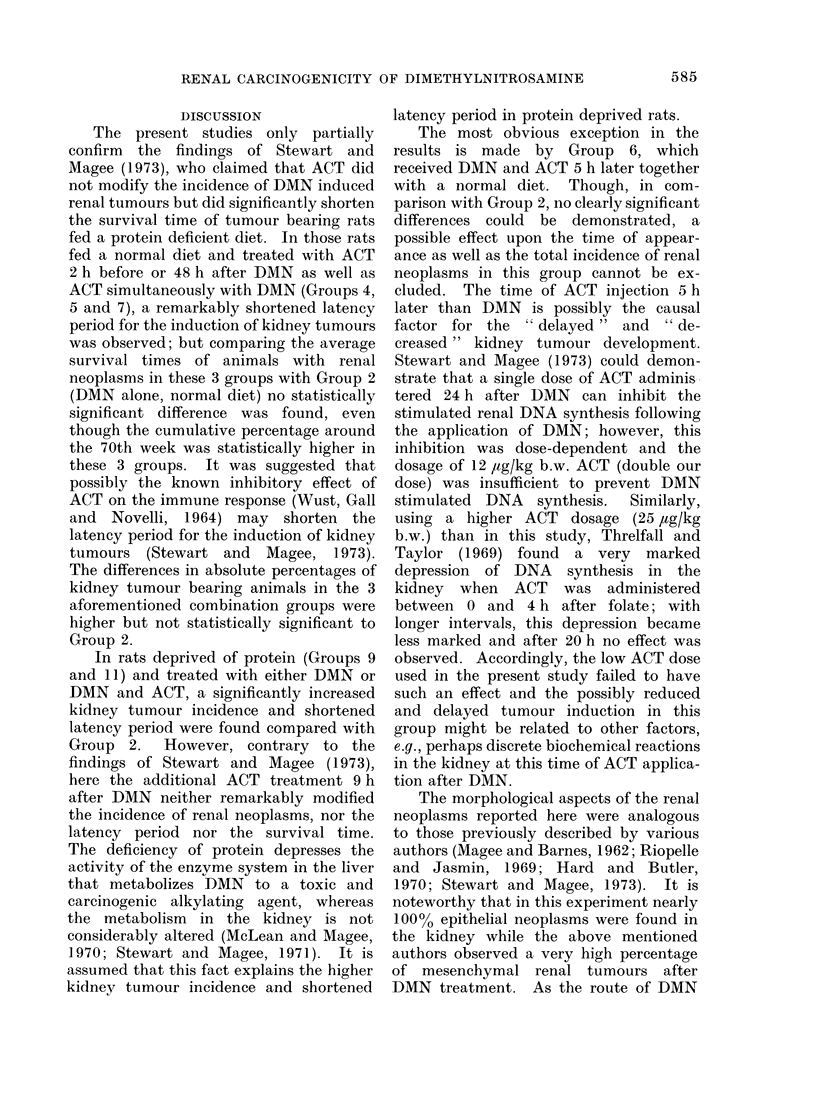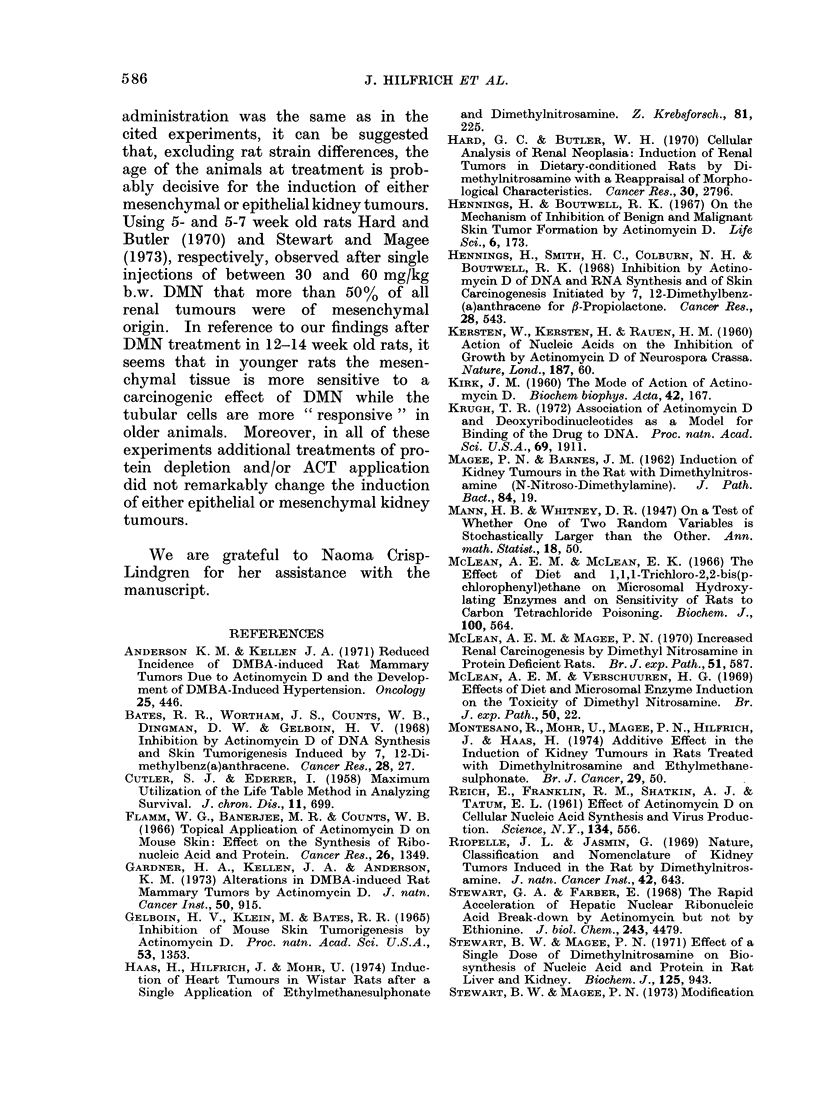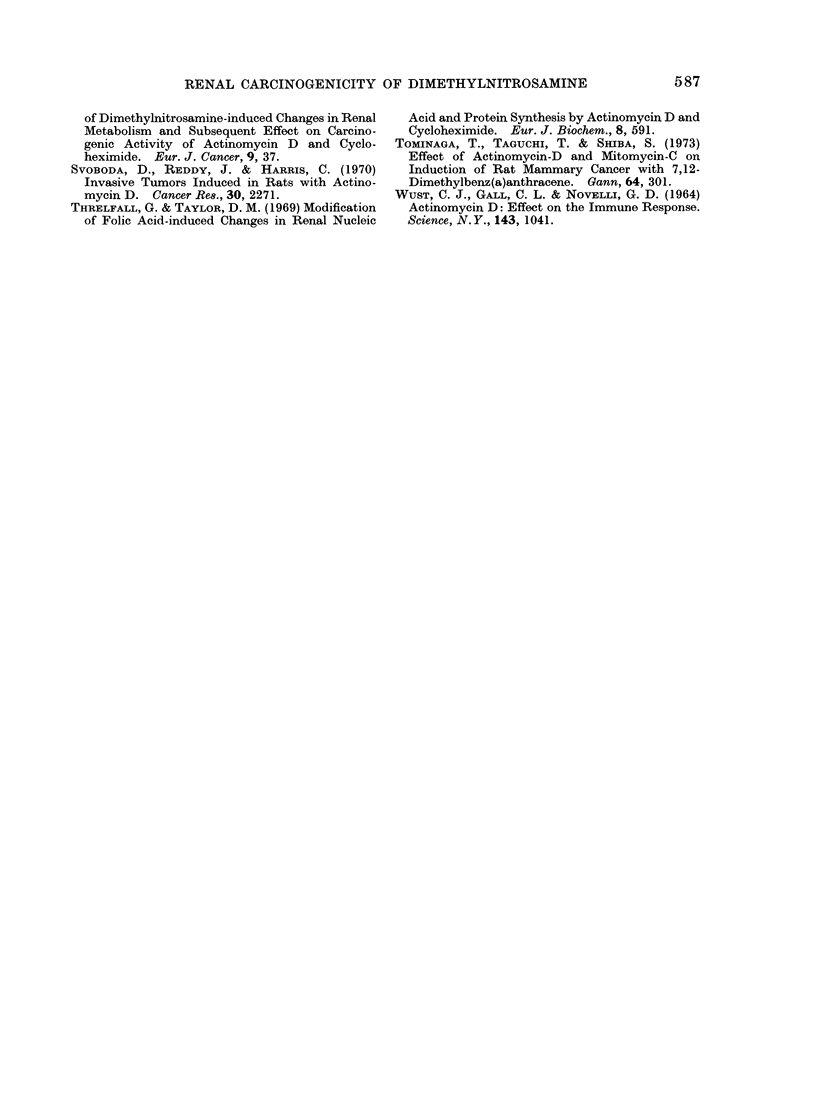# The modification of the renal carcinogenicity of dimethylnitrosamine by actinomycin D and a protein deficient diet.

**DOI:** 10.1038/bjc.1975.264

**Published:** 1975-11

**Authors:** J. Hilfrich, H. Haas, N. Kmoch, R. Montesano, U. Mohr, P. N. Magee

## Abstract

**Images:**


Br. J. Cancer (1975) **32**, 578

### THE MODIFICATION OF THE RENAL CARCINOGENICITY OF DIMETHYLNITROSAMINE BY ACTINOMYCIN D AND A PROTEIN DEFICIENT DIET*

J. HILFRICH, H. HAAS, N. KMOCH, R. MONTESANO†, U. MOHR AND P. N. MAGEE‡

*From the Abteilung für Experimentelle Pathologie, Medizinische Hochschule Hannover, 3000 Hannover-Kleefeld, Karl-Wiechert-Allée 9, FRG, the †Unit of Chemical Carcinogenesis, International Agency for Research on Cancer, 150, Cours Albert Thomas, 69008 Lyon, France, and the ‡Courtauld Institute of Biochemistry, The Middlesex Hospital Medical School, London, W1P 5PR, England*

Received 9 June 1975. Accepted 30 July 1975

**Summary.—The effect of a single treatment with 30 mg dimethylnitrosamine (DMN) and 6 μg actinomycin D (ACT), given at different time intervals (ACT application to DMN, 2 h before, simultaneously, 5, 9 or 48 h later), was tested in female Sprague–Dawley rats in relation to renal carcinogenesis; additionally, the animals were fed either a normal or a protein deficient diet.**

**The ACT treatment did not significantly modify either the kidney tumour incidence or the survival time in the different groups fed a normal diet. Nevertheless, there are indications that additional ACT application may shorten the latency period for DMN induced renal neoplasms or, when administered 5 h later than DMN, a slightly decreased and delayed tumour induction can be assumed. In groups fed a protein deficient diet, a significantly higher percentage of kidney tumour bearing animals as well as a shortened latency period were found when compared with the DMN group on normal diet, but these differences were independent of the additional ACT treatment 9 h later than DMN and were due to the protein deprivation. Morphologically, the tumours were of epithelial and mesenchymal type with a clear preponderance of the former type. Biochemical and morphological aspects are discussed.**

THE DEVELOPMENT of tumours in some animal species exposed to carcinogenic substances may be modified by secondary treatments. The antibiotic actinomycin D (ACT), which can bind to DNA and inhibit the DNA dependent RNA synthesis (Kirk, 1960; Kersten, Kersten and Rauen, 1960; Reich *et al.*, 1961; Flamm, Banerjee and Counts, 1966; Stewart and Farber, 1968; Bates *et al.*, 1968; Hennings *et al.*, 1968; Threlfall and Taylor, 1969; Krugh, 1972), inhibits the induction of skin tumours in mice by 7,12-dimethylbenz(a)anthracene (DMBA) (Gelboin, Klein and Bates, 1965; Hennings and Boutwell, 1967; Bates *et al.*, 1968) and also reduces the incidence of mammary neoplasms in rats induced by the same compound (Anderson and Kellen, 1971; Gardner, Kellen and Anderson, 1973; Tominaga, Taguchi and Shiba, 1973). Stewart and Magee (1973), however, have shown that in protein depleted rats ACT did not modify the incidence of dimethylnitrosamine (DMN) induced renal tumours but did affect the survival time of rats bearing the tumours.

As a continuation of this experiment, the effect of a single treatment with DMN and ACT in different combinations was studied in rats with regard to renal carcinogenesis. Additionally, either a normal or a protein deficient diet was fed since protein depletion is known to in-

* Presented in part at the XIth International Cancer Congress, Florence, Italy, October, 1974.

fluence the toxicity and the carcinogenic effect of DMN by inhibition of the drug metabolizing enzymes in the liver (McLean and Verschuuren, 1969; McLean and Magee, 1970).

### MATERIALS AND METHODS

Three hundred and thirty female Sprague-Dawley rats (Wiga, Sulzfeld, FRG) 12–14 weeks old were divided into 11 equal groups of 30 animals and housed 5 rats per Makrolon cage, Type III (E. Becker and Co., Castrop-Rauxel, FRG) under standard laboratory conditions (temperature, 22 ± 2°C; relative humidity, 55 ± 5%; air exchange, 8 times/h) and given water *ad libitum*. The treatment groups are listed in Table I. Groups 1–3 were treated i.p. with 1 ml saline/kg body weight (b.w.) as vehicle controls, 30 mg DMN (synthesized by F. W. Krüger, DKFZ, Heidelberg, purity, 99·6%) per kg b.w. dissolved in saline or 6 μg ACT (Sigma Chemicals, USA) per kg b.w. alone; Groups 4–7 received the treatment combinations of DMN and ACT and were fed as Groups 1–3 with a normal diet (Hope Farms, RMH-TMB, Woerden, Holland); in Group 4, ACT was given 2 h before DMN, in Groups 5, 6 and 7, ACT was injected simultaneously 5 h or 48 h after DMN. In Groups 8–11 the treatment with saline, DMN and ACT alone or DMN and ACT 9 h later were combined with a protein deficient diet (Hope Farms RMH-TMB/McLean and McLean, 1966). The diet was given 1 week before and 1 week after treatment.

All animals died spontaneously or were killed when moribund. Except for a few animals lost through cannibalism, all were completely autopsied, the organs fixed in 4% buffered formalin and processed for routine histological examination. Eight graded histological sections were made from each kidney. The effective number of animals (Table I) is based on the number of rats surviving after the first tumour of any site (28 weeks) had been observed. For statistical evaluation according to the method of Cutler and Ederer (1958), the percentage of kidney tumour bearing animals was calculated in 4-week intervals and cumulated (cumulative per cent, Fig. 1, 2). For statistical comparison of the kidney tumour bearing animals in the different groups, the chi-square test, and for the incidence of kidney tumours per animal as well as the average survival times in the various groups, the U-test after Mann and Whitney (1947) were performed.

### RESULTS

Table I summarizes information on the tumour bearing animals, the average survival rates as well as the tumour incidence of all organs in the different treatment groups.

No kidney tumour was observed in saline treated control animals either with normal or protein deficient diet (Groups 1 and 8) and after treatment with ACT and normal diet (Group 3). In Group 10 (ACT, protein deficient diet) only one kidney neoplasm was diagnosed after 120 weeks. In all other DMN treated groups, numerous renal tumours were found. Moreover, tumours of organs other than the kidney were detected, mainly of the mammary gland (fibromata, fibrosarcomata, fibroadenomata, carcinomata and a few carcinosarcomata), the lymphatic system (malignant lymphomata, reticulosarcomata and thymomata), the nasal and paranasal cavities (papillomata, squamous and adenocarcinomata, esthesioneuroepitheliomata), the heart (neurofibromata and one neurofibrosarcoma), the adrenal gland (cortical adenomata) as well as other various organs (Table I). Average survival times and tumour incidence in Group 3 (ACT, normal diet), Group 8 (NaCl, protein deficient diet) and Group 10 (ACT, protein deficient diet) are comparable with the control Group 1 (NaCl, normal diet), except for the occurrence of one kidney (tubular carcinoma) and one coronary (neurofibrosarcoma) tumour in Group 10, usually seen only in the DMN treated groups. The dose of ACT used in the present experiment was much less than that required to produce acute toxic symptoms or to induce tumours (Svoboda, Reddy and Harris, 1970).

In Table II the detailed results of

TABLE I.—*Tumour Incidence in Female Sprague–Dawley Rats after Treatment with DMN, ACT and Protein Deficient Diet in Different Combinations*

* Total tumour bearing animals.

‡ The protein deficient diet was given 1 week before and 1 week after treatment.

TABLE II.—*Kidney Tumours Induced in Female Sprague–Dawley Rats after Treatment with DMN, ACT and Protein Deficient Diet in Different Combinations*

| Treatment groups (dosage per kg b.w., i.p.) | TBA* (%) | Average survival of TBA in weeks (range) | Bilateral TBA | Total no. of kidney tumours | Ratio | Kidney tumours | |
|---|---|---|---|---|---|---|---|
| | | | | | | Epithelial | Mesenchymal |
| 1. NaCl (control) | — | — | — | — | — | — | — |
| 2. DMN | 10 (43·4) | 94·5 (74–113) | 4 | 21 | 2·10 | 21 | — |
| 3. ACT | — | — | — | — | — | — | — |
| 4. ACT + DMN (2 h) | 14 (58·3) | 81·5 (48–107) | 7 | 24 | 1·71 | 24 | — |
| 5. DMN + ACT (simult.) | 13 (56·5) | 86·4 (48–127) | 3 | 21 | 1·62 | 18 | 3 |
| 6. DMN + ACT (5 h) | 8 (30·8) | 100·1 (73–128) | 2 | 13 | 1·63 | 13 | — |
| 7. DMN + ACT (48 h) | 15 (57·7) | 85·9 (42–120) | 7 | 26 | 1·73 | 23 | 3 |
| 8. NaCl + prot. def. diet | — | — | — | — | — | — | — |
| 9. DMN + prot. def. diet | 30 (100) | 69·8 (47–96) | 26 | 93 | 3·10 | 88 | 5 |
| 10. ACT + prot. def. diet | 1 (3·8) | 120 | — | 1 | 1·00 | 1 | — |
| 11. DMN + ACT (9 h) + prot. def. diet | 24 (92·3) | 65·4 (33–118) | 17 | 61 | 2·54 | 54 | 7 |

* Kidney tumour bearing animals.

kidney tumour incidence in the different groups are listed. The renal neoplasms, which occurred bilaterally in a certain number of animals, showed a clear preponderance of epithelial over mesenchymal tumours in all groups.

Comparing the percentage of kidney tumour bearing animals of Group 2 (DMN, normal diet) with those in each other group treated with DMN, ACT and normal diet (Groups 4–7), no significant difference ($P > 0{\cdot}05$) was found. However, in Groups 9 and 11 (DMN or DMN and ACT with protein deficient diet), the incidence of 100% and 92·8%, respectively, of kidney tumour bearing animals was significantly increased ($P < 0{\cdot}01$) compared with Group 2.

Since comparison of the number or percentage of tumour bearing animals based on the initial number of rats can be misleading if deaths due to causes other than the observed tumours occurred at different rates in the various experimental groups, the cumulative percentage for rats with renal neoplasms has been calculated in 4-week intervals (Cutler and Ederer, 1958). In Fig. 1 the results for Group 2 (DMN, normal diet) and Groups 4–7 (combined treatment of DMN and ACT, normal diet) are plotted. While in Group 2 the first renal neoplasm was seen in the 76th week, in Groups 4, 5 and 7 the first renal tumours appeared as early as in the 44th or 52nd week after beginning treatment. For the period from the 64th until the 72nd week, these 3 groups demonstrated a significantly higher cumulative percentage of rats with renal neoplasms compared with Group 2. An exception to the 3 aforementioned combination groups was made by Group 6 (DMN, ACT 5 h later). In this group, the first tumour was seen at the same time as in Group 2 but subsequently the cumulative percentages are remarkably lower than in the DMN Group 2. This difference was statistically significant only for the period from 112 to 116th weeks of the survival time; however, for the periods from 92nd to 96th and 104th to 112th weeks borderline values ($0{\cdot}1 > P > 0{\cdot}05$) were found.

The shortened latency period for induction of kidney tumours in Groups 4, 5 and 7 was not statistically significant ($P > 0{\cdot}1$) in comparison with Group 2.

In Fig. 2 Groups 9 and 11 (which received protein deficient diet and DMN or DMN and ACT 9 h later) are plotted in relation to Group 2. For the period from the 52nd (Group 9) or 44th week (Group 11) until the 104th (Group 9) and 100th (Group 11) of the survival time, significant differences between the cumulative percentages were found. Here, the shortened latency period for renal tumour induction was highly significant ($0{\cdot}001 < P < 0{\cdot}002$).

FIG. 1.—Cumulative percentage of kidney tumour bearing animals in relation to the survival time after treatment with DMN alone or DMN and ACT in different combinations; these rats received a normal diet.

Differences in kidney tumour incidences per animal in Groups 4–7 as well as 9 and 11 failed to be statistically significant when compared with the incidence per animal in the DMN Group 2. Only for Group 6 (DMN, ACT 5 h later; normal diet) was a borderline value ($0{\cdot}1 > P > 0{\cdot}05$) obtained.

Macroscopically, the induced kidney tumours showed mostly grey–white nodules (Fig. 3), increasing in size with a tendency to necroses and haemorrhages. In one kidney up to 5 tumours were found. Pulmonary metastases were observed only rarely. Histologically, the renal neoplasms were predominantly tubular adenomata and carcinomata with expansive growth (Fig. 4); in a few cases, mesenchymal tumours could be diagnosed (Fig. 5). The morphological patterns of the renal neoplasms were similar for all groups.

Among the other types of tumours, marked differences were observed only for nasal cavity and heart tumours, which occurred mainly in those groups receiving DMN and ACT in different combinations (Groups 4, 5, 6, 9 and 11). Since a single dosage of 30 mg DMN can induce also nasal cavity (Montesano *et al.*, 1974) and neurogenic coronary tumours (Haas, Hilfrich and Mohr, 1974), it seems that in relation to the average survival time, only the induction of nasal cavity neoplasms in Group 11 (DMN, ACT and protein deficient diet) was slightly increased (Table I).

Fig. 2.—Cumulative percentage of kidney tumour bearing animals in relation to the survival time after treatment with DMN or DMN and ACT as well as a protein deficient diet. For comparison, additionally Group 2, DMN alone, normal diet, is plotted.

Fig. 3.—Bilateral multiple renal tumours (53 weeks, DMN and protein deficient diet). ×1·6.

FIG. 4.—Part of a tubular kidney carcinoma with expansive growth. H. and E. ×120.

FIG. 5.—Mesenchymal neoplasm of the kidney, within the tumour tissue a retained and not extensively affected glomerulum. H. and E. ×340.

### DISCUSSION

The present studies only partially confirm the findings of Stewart and Magee (1973), who claimed that ACT did not modify the incidence of DMN induced renal tumours but did significantly shorten the survival time of tumour bearing rats fed a protein deficient diet. In those rats fed a normal diet and treated with ACT 2 h before or 48 h after DMN as well as ACT simultaneously with DMN (Groups 4, 5 and 7), a remarkably shortened latency period for the induction of kidney tumours was observed; but comparing the average survival times of animals with renal neoplasms in these 3 groups with Group 2 (DMN alone, normal diet) no statistically significant difference was found, even though the cumulative percentage around the 70th week was statistically higher in these 3 groups. It was suggested that possibly the known inhibitory effect of ACT on the immune response (Wust, Gall and Novelli, 1964) may shorten the latency period for the induction of kidney tumours (Stewart and Magee, 1973). The differences in absolute percentages of kidney tumour bearing animals in the 3 aforementioned combination groups were higher but not statistically significant to Group 2.

In rats deprived of protein (Groups 9 and 11) and treated with either DMN or DMN and ACT, a significantly increased kidney tumour incidence and shortened latency period were found compared with Group 2. However, contrary to the findings of Stewart and Magee (1973), here the additional ACT treatment 9 h after DMN neither remarkably modified the incidence of renal neoplasms, nor the latency period nor the survival time. The deficiency of protein depresses the activity of the enzyme system in the liver that metabolizes DMN to a toxic and carcinogenic alkylating agent, whereas the metabolism in the kidney is not considerably altered (McLean and Magee, 1970; Stewart and Magee, 1971). It is assumed that this fact explains the higher kidney tumour incidence and shortened latency period in protein deprived rats.

The most obvious exception in the results is made by Group 6, which received DMN and ACT 5 h later together with a normal diet. Though, in comparison with Group 2, no clearly significant differences could be demonstrated, a possible effect upon the time of appearance as well as the total incidence of renal neoplasms in this group cannot be excluded. The time of ACT injection 5 h later than DMN is possibly the causal factor for the "delayed" and "decreased" kidney tumour development. Stewart and Magee (1973) could demonstrate that a single dose of ACT administered 24 h after DMN can inhibit the stimulated renal DNA synthesis following the application of DMN; however, this inhibition was dose-dependent and the dosage of 12 $\mu$g/kg b.w. ACT (double our dose) was insufficient to prevent DMN stimulated DNA synthesis. Similarly, using a higher ACT dosage (25 $\mu$g/kg b.w.) than in this study, Threlfall and Taylor (1969) found a very marked depression of DNA synthesis in the kidney when ACT was administered between 0 and 4 h after folate; with longer intervals, this depression became less marked and after 20 h no effect was observed. Accordingly, the low ACT dose used in the present study failed to have such an effect and the possibly reduced and delayed tumour induction in this group might be related to other factors, *e.g.*, perhaps discrete biochemical reactions in the kidney at this time of ACT application after DMN.

The morphological aspects of the renal neoplasms reported here were analogous to those previously described by various authors (Magee and Barnes, 1962; Riopelle and Jasmin, 1969; Hard and Butler, 1970; Stewart and Magee, 1973). It is noteworthy that in this experiment nearly 100% epithelial neoplasms were found in the kidney while the above mentioned authors observed a very high percentage of mesenchymal renal tumours after DMN treatment. As the route of DMN

administration was the same as in the cited experiments, it can be suggested that, excluding rat strain differences, the age of the animals at treatment is probably decisive for the induction of either mesenchymal or epithelial kidney tumours. Using 5- and 5-7 week old rats Hard and Butler (1970) and Stewart and Magee (1973), respectively, observed after single injections of between 30 and 60 mg/kg b.w. DMN that more than 50% of all renal tumours were of mesenchymal origin. In reference to our findings after DMN treatment in 12–14 week old rats, it seems that in younger rats the mesenchymal tissue is more sensitive to a carcinogenic effect of DMN while the tubular cells are more " responsive " in older animals. Moreover, in all of these experiments additional treatments of protein depletion and/or ACT application did not remarkably change the induction of either epithelial or mesenchymal kidney tumours.

We are grateful to Naoma Crisp-Lindgren for her assistance with the manuscript.

REFERENCES

ANDERSON K. M. & KELLEN J. A. (1971) Reduced Incidence of DMBA-induced Rat Mammary Tumors Due to Actinomycin D and the Development of DMBA-Induced Hypertension. *Oncology* **25**, 446.

BATES, R. R., WORTHAM, J. S., COUNTS, W. B., DINGMAN, D. W. & GELBOIN, H. V. (1968) Inhibition by Actinomycin D of DNA Synthesis and Skin Tumorigenesis Induced by 7, 12-Dimethylbenz(a)anthracene. *Cancer Res.*, **28**, 27.

CUTLER, S. J. & EDERER, I. (1958) Maximum Utilization of the Life Table Method in Analyzing Survival. *J. chron. Dis.*, **11**, 699.

FLAMM, W. G., BANERJEE, M. R. & COUNTS, W. B. (1966) Topical Application of Actinomycin D on Mouse Skin: Effect on the Synthesis of Ribonucleic Acid and Protein. *Cancer Res.*, **26**, 1349.

GARDNER, H. A., KELLEN, J. A. & ANDERSON, K. M. (1973) Alterations in DMBA-induced Rat Mammary Tumors by Actinomycin D. *J. natn. Cancer Inst.*, **50**, 915.

GELBOIN, H. V., KLEIN, M. & BATES, R. R. (1965) Inhibition of Mouse Skin Tumorigenesis by Actinomycin D. *Proc. natn. Acad. Sci. U.S.A.*, **53**, 1353.

HAAS, H., HILFRICH, J. & MOHR, U. (1974) Induction of Heart Tumours in Wistar Rats after a Single Application of Ethylmethanesulphonate and Dimethylnitrosamine. *Z. Krebsforsch.*, **81**, 225.

HARD, G. C. & BUTLER, W. H. (1970) Cellular Analysis of Renal Neoplasia: Induction of Renal Tumors in Dietary-conditioned Rats by Dimethylnitrosamine with a Reappraisal of Morphological Characteristics. *Cancer Res.*, **30**, 2796.

HENNINGS, H. & BOUTWELL, R. K. (1967) On the Mechanism of Inhibition of Benign and Malignant Skin Tumor Formation by Actinomycin D. *Life Sci.*, **6**, 173.

HENNINGS, H., SMITH, H. C., COLBURN, N. H. & BOUTWELL, R. K. (1968) Inhibition by Actinomycin D of DNA and RNA Synthesis and of Skin Carcinogenesis Initiated by 7, 12-Dimethylbenz-(a)anthracene for β-Propiolactone. *Cancer Res.*, **28**, 543.

KERSTEN, W., KERSTEN, H. & RAUEN, H. M. (1960) Action of Nucleic Acids on the Inhibition of Growth by Actinomycin D of Neurospora Crassa. *Nature, Lond.*, **187**, 60.

KIRK, J. M. (1960) The Mode of Action of Actinomycin D. *Biochem biophys. Acta*, **42**, 167.

KRUGH, T. R. (1972) Association of Actinomycin D and Deoxyribodinucleotides as a Model for Binding of the Drug to DNA. *Proc. natn. Acad. Sci. U.S.A.*, **69**, 1911.

MAGEE, P. N. & BARNES, J. M. (1962) Induction of Kidney Tumours in the Rat with Dimethylnitrosamine (N-Nitroso-Dimethylamine). *J. Path. Bact.*, **84**, 19.

MANN, H. B. & WHITNEY, D. R. (1947) On a Test of Whether One of Two Random Variables is Stochastically Larger than the Other. *Ann. math. Statist.*, **18**, 50.

McLEAN, A. E. M. & McLEAN, E. K. (1966) The Effect of Diet and 1,1,1-Trichloro-2,2-bis(p-chlorophenyl)ethane on Microsomal Hydroxylating Enzymes and on Sensitivity of Rats to Carbon Tetrachloride Poisoning. *Biochem. J.*, **100**, 564.

McLEAN, A. E. M. & MAGEE, P. N. (1970) Increased Renal Carcinogenesis by Dimethyl Nitrosamine in Protein Deficient Rats. *Br. J. exp. Path.*, **51**, 587.

McLEAN, A. E. M. & VERSCHUUREN, H. G. (1969) Effects of Diet and Microsomal Enzyme Induction on the Toxicity of Dimethyl Nitrosamine. *Br. J. exp. Path.*, **50**, 22.

MONTESANO, R., MOHR, U., MAGEE, P. N., HILFRICH, J. & HAAS, H. (1974) Additive Effect in the Induction of Kidney Tumours in Rats Treated with Dimethylnitrosamine and Ethylmethanesulphonate. *Br. J. Cancer*, **29**, 50.

REICH, E., FRANKLIN, R. M., SHATKIN, A. J. & TATUM, E. L. (1961) Effect of Actinomycin D on Cellular Nucleic Acid Synthesis and Virus Production. *Science, N.Y.*, **134**, 556.

RIOPELLE, J. L. & JASMIN, G. (1969) Nature, Classification and Nomenclature of Kidney Tumors Induced in the Rat by Dimethylnitrosamine. *J. natn. Cancer Inst.*, **42**, 643.

STEWART, G. A. & FARBER, E. (1968) The Rapid Acceleration of Hepatic Nuclear Ribonucleic Acid Break-down by Actinomycin but not by Ethionine. *J. biol. Chem.*, **243**, 4479.

STEWART, B. W. & MAGEE, P. N. (1971) Effect of a Single Dose of Dimethylnitrosamine on Biosynthesis of Nucleic Acid and Protein in Rat Liver and Kidney. *Biochem. J.*, **125**, 943.

STEWART, B. W. & MAGEE, P. N. (1973) Modification of Dimethylnitrosamine-induced Changes in Renal Metabolism and Subsequent Effect on Carcinogenic Activity of Actinomycin D and Cycloheximide. *Eur. J. Cancer*, **9**, 37.

SVOBODA, D., REDDY, J. & HARRIS, C. (1970) Invasive Tumors Induced in Rats with Actinomycin D. *Cancer Res.*, **30**, 2271.

THRELFALL, G. & TAYLOR, D. M. (1969) Modification of Folic Acid-induced Changes in Renal Nucleic Acid and Protein Synthesis by Actinomycin D and Cycloheximide. *Eur. J. Biochem.*, **8**, 591.

TOMINAGA, T., TAGUCHI, T. & SHIBA, S. (1973) Effect of Actinomycin-D and Mitomycin-C on Induction of Rat Mammary Cancer with 7,12-Dimethylbenz(a)anthracene. *Gann*, **64**, 301.

WUST, C. J., GALL, C. L. & NOVELLI, G. D. (1964) Actinomycin D: Effect on the Immune Response. *Science, N.Y.*, **143**, 1041.